# Fatal adult-onset diaphragmatic hernia in the context of the COVID-19 pandemic

**DOI:** 10.4322/acr.2021.366

**Published:** 2022-01-14

**Authors:** Ajith Antony, Sheryl Suares, André Victor Fernandes

**Affiliations:** 1 Goa Medical College, Department of Forensic Medicine and Toxicology, Bambolim, Goa, India

**Keywords:** Hernias, Diaphragmatic, Congenital, COVID-19, Genetics, case report

## Abstract

Late-presenting or “Adult-Onset” Diaphragmatic Hernia is uncommon, especially in individuals with no history of trauma. The non-traumatic diaphragmatic hernia may be a Congenital Diaphragmatic Hernia [CDH] lately manifested as a sequela to an iatrogenic intervention or prolonged infections. We aim to explore the genetic correlations in “adult-onset” CDH, with an insight into the indirect contribution of the COVID-19 pandemic towards the fatal outcome.In this report, we present a case of an adult female who died from the complications of an undiagnosed adult-onset diaphragmatic hernia, deemed completely preventable, if not for the global COVID-19 pandemic. There was no prior history of physical trauma or medical history of any relevance.Early diagnosis and rapid surgical intervention remain the keystone management for successfully treating individuals affected by this entity. The decedent in question presented with symptoms demanding hospital stay for investigations that would have aided in timely diagnosis and prevented death. However, the excessive fear of COVID-19 prevented the patient from undergoing hospitalization and follow-up, delaying the diagnosis and leading to death.

## INTRODUCTION

Diaphragmatic hernias can be congenital, occurring in about one out of every 3600 live births in the United States,^1^^,^ 2 or acquired. A non-traumatic etiology causing an “adult-onset” diaphragmatic hernia can be due to a delayed presentation of a congenital diaphragmatic hernia [CDH], or as a consequence of iatrogenic procedures, especially surgical interventions, or as delayed sequelae to prolonged infection. Out of the CDHs presenting in the adult age group, most are associated with trauma.^3^

In adults, sudden death consequent to an undiagnosed CDH is rare.^4^^,^ 5 Early diagnosis and prompt surgical intervention remain the key to efficient management in such cases. However, owing to the non-specific nature of symptoms, the subtlety of the clinical signs and the overall rapid onset, a prompt diagnosis by direct suspicion is needed. A delayed diagnosis is usually the culprit behind a fatal outcome.

We report a case of a young female patient who died of non-traumatic, “adult-onset” diaphragmatic hernia, undiagnosed owing to the dynamics of the COVID-19 pandemic, along with a review of genetic predilections that lead to this condition.

## CASE REPORT

A 27-year-old female sought the outpatient department of a primary health center complaining of recent-onset abdominal pain, vomiting, and bowel disturbances. She had two similar episodes in the past 3 months, but it subsided without medical intervention. The patient presented with these severe symptoms during the nationwide lockdown imposed by the Central Government after the invocation of section 6, Disaster Management Act. Physical examination and radiological examination of the abdomen was done during the earlier presentation; however, the same proved to be unremarkable. Hence, she was managed conservatively with gastritis as the most likely diagnosis.

Two days later, she presented again with severe epigastric pain followed by eight episodes of vomiting, fever, and breathlessness. Routine blood tests and ECG were normal. Along with the regular workup for evaluating the fever, gastrointestinal complaints and breathlessness, the prevalent pandemic conditions alerted the resident doctor at the primary health center to order a COVID-19 test. Swabs for COVID-19 tests were taken, and reports were awaited. Doctors with a basic medical degree usually oversee the Primary Healthcare Centers (PHCs) in India, referring cases needing detailed workup to a Tertiary Healthcare Center. The PHCs usually lack facilities like laboratories and radiological equipment. Even though the treating doctor referred her to a tertiary care center and advised admission, she refused to stay in hospital premises, fearing COVID-19 pandemic, and demanded conservative management.

The treating doctor also advised her to undergo a repeat-radiological examination of the chest, abdomen, and pelvis and emphasized isolation. Again, fearing the pandemic and rules of compulsory isolation, the patient refused to acknowledge the referral and took discharge against medical advice. Soon, she became extremely dyspneic and expired at home. Her COVID-19 RT-PCR reports turned out to be negative.

## AUTOPSY PRESENTATION

**Figure 1:**
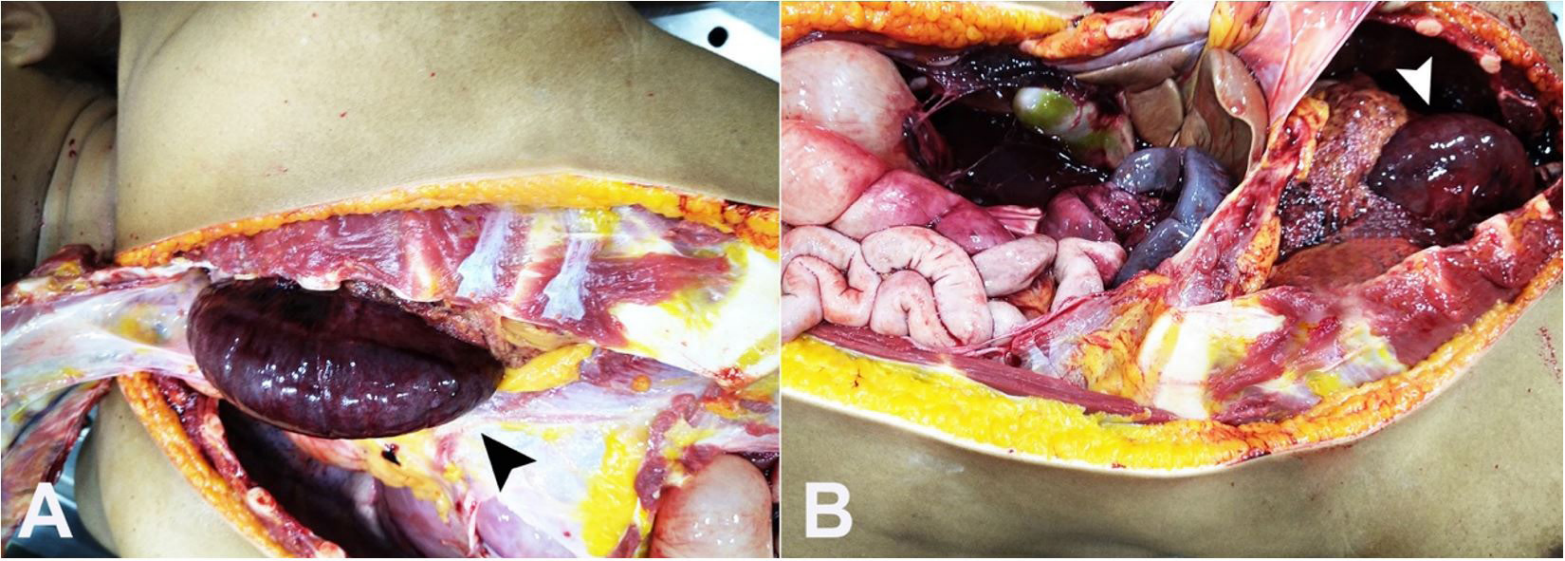
Gross findings after the thoracoabdominal cavities overture. **A** – A loop of gangrenous small intestine seen upon opening the ribcage (black arrowhead); **B** – Left hemidiaphragm showing a defect with smooth edges, through which the omentum, the gastric fundus and a loop of gangrenous small intestine (white arrowhead) are seen herniating into the left thoracic cavity.

The decedent was autopsied five hours after her death. External examination was unremarkable, with neither injuries nor abnormalities. She had fused earlobes, with relatively small fingers and a short stature [body length=148 cm]. Internal examination revealed a loop of gangrenous small intestine upon opening the ribcage (Figure 1A). Further dissection revealed a defect in the left hemidiaphragm measuring 6 cm in length and 4 cm in breadth. This defect had smooth edges, with the omentum, the gastric fundus and a loop of gangrenous small intestine herniating into the left thoracic cavity (Figure 1B). Both lungs were edematous and congested. No sign of excessive fluid was present in the pleural, pericardial, or peritoneal cavities. Other organs showed congestive and edematous features. The final cause of death was opined as septicemia consequent to gangrene of the strangulated small intestine in a person with adult-onset diaphragmatic hernia.

Although her external appearance with a non-traumatic etiology of diaphragmatic hernia warranted a genetic evaluation, neither the institution nor the medico-legal system had the necessary provisions to carry out a genetic study. The family was advised to do the same in a private set up to rule out any inheritable genetic diseases. The family refused to do the test, citing financial constraints.

## DISCUSSION

Diaphragmatic hernia refers to a defect in the diaphragm that allows the abdominal contents to migrate into the thoracic cavity. Such defects are common after sustaining blunt or penetrating trauma to the chest and abdomen,^6^^,^ 7 especially following high-velocity impact in road traffic accidents.^8^ Left postero-lateral herniation is more common, owing to the inherent weakness of the embryonic fusion lines of the pleuroperitoneal membrane on the left side and a mechanical barrier formed by the liver on the right side.^7^ In the present case, there were no visible signs of trauma, or a suggestive history.

In the absence of trauma, a diaphragmatic hernia is a rarity in adults, usually preceded by a weakening esophageal hiatus leading to hiatal hernias. Even more unusual is when a CDH manifests in adulthood in non-traumatic conditions. CDH occurs consequent to incomplete fusion of pleuroperitoneal folds during the first trimester of gestation,^9^ consequent to a plethora of genetic causes, of which about 70% has an unknown etiology.^10^ Epidemiological studies^11^^,^ 12 on congenital diaphragmatic hernias reported a frequency of 1 in 2500 to 1 in 3715 live births, with about 28% of infants suffering from other congenital anomalies, recording a strong association with Trisomy 18 as well as neural tube defects.

The genetic etiology of CDH could be related to a single gene, with the mode of inheritance being either autosomal dominant (Table 1), autosomal recessive (Table 2) or X-linked (Table 3), or chromosome-related as shown in Table 4.^10^^,^ 13 ^-^ 20 Sometimes, a combination of both may occur in certain syndromic presentations [e.g., Beckwith-Wiedemann syndrome], or the genetic etiology could have an unknown mode of inheritance [e.g., Fryns syndrome, Pentalogy of Cantrell, Thoracoabdominal syndrome].

**Table 1:** Syndromes associated with CDH with an Autosomal Dominant mode of inheritance

Gene[s]	Syndrome	Predilection
*MYRF*	Cardiac-urogenital syndrome	High
*NR2F2*	Congenital heart defects, multiple types, 4	Moderate
*ZFPM2*	Diaphragmatic hernia type 3	Moderate
*ARID1A*	Coffin-Siris syndrome	Low
*ARID1B*		
*SMARCA4*		
*SMARCB1*		
*SMARCE1*		
*SOX11*		
*CHD7*	CHARGE syndrome	Low
*COL3A1*	Vascular Ehlers-Danlos syndrome	Low
*FBN1*	Marfan syndrome	Low
*FGFR2*	Apert syndrome	Low
*GATA4*	Congenital diaphragmatic hernia & heart defects	Low
*GATA6*	Heart defects, other congenital anomalies	Low
*POGZ*	White-Sutton syndrome	Low
*WT1*	Denys-Drash syndrome	Low
	Meacham syndrome	
*KMT2D*	Kabuki syndrome	Low
*NIPBL*	Cornelia de Lange syndrome	Low
*RAD21*		
*SMC3*		
Imprinting defect at *11p15.5*	Beckwith-Wiedemann syndrome	Low
*CDKN1C*		

**Table 2:** Syndromes associated with CDH with an Autosomal Recessive mode of inheritance

Gene[s]	Syndrome	Predilection
*LRP2*	Donnai-Barrow syndrome	High
*LTBP4*	LTBP4-related cutis laxa	High
*RARB*	Microphthalmia, syndromic	High
*STRA6*		
*PIGN*	PIGN-related Fryns syndrome 3	High
*Unknown*	Fryns syndrome	High
*SLC2A10*	Arterial tortuosity syndrome	Moderate
*DLL3*	Spondylocostal dysostosis	Low
*HES7*		
*LFNG*		
*MESP2*		
*RIPPLY2*		
*TBX6*		
*FRAS1*	Fraser syndrome	Low
*FREM2*		

**Table 3:** Syndromes associated with CDH with X-Linked mode of inheritance

Gene[s]	Syndrome	Predilection
*RLIM*	Tonne-Kalscheuer syndrome	High
*Unknown*	Pentalogy of Cantrell	High
*COX7B*	Microphthalmia with linear skin defects syndrome	Moderate
*HCCS*		
*NDUFB11*		
*Unknown*	Thoracoabdominal syndrome^#^	Moderate
*EFNB1*	Craniofrontonasal syndrome	Low
*PORCN*	Focal dermal hypoplasia	Low
*GPC3*	Simpson-Golabi-Behmel syndrome type 1	Low
*KDM6A*	Kabuki syndrome	Low
*HDAC8*	Cornelia de Lange syndrome	Low
*SMC1A*		

**Table 4:** Syndromes associated with CDH with Chromosomal mode of inheritance

**Chromosome / Locus**	**Syndrome**	**Predilection**
mosaic tetrasomy 12p	Pallister-Killian syndrome	Moderate
Del 1q41-q42	1q41q42 microdeletion syndrome	Moderate
Del 4p16.3	Wolf-Hirschhorn syndrome	Moderate
Del 8p23.1	8p23.1 microdeletion syndrome	Moderate
Del 15q24	15q24 Microdeletion syndrome	Moderate
Del 15q26.2	Drayer's syndrome/ recurrent deletion of chromosome 15[q26.2→qter]	Moderate
Del 17q12	Chromosome 17q12 deletion syndrome	Moderate
Del 8q23.1	Langer-Giedion syndrome [LGS]	Low
Del 22q11	DiGeorge syndrome	Low
+der [22] t[11;22][q23;q11]	Emanuel syndrome/ Supernumerary der[22]t[11;22] syndrome	Low
Trisomy 13	Patau’s Syndrome	Low
Trisomy 18	Edward’s Syndrome	Low
Trisomy 21	Down’s Syndrome [Morgagni hernias > Bochdalek hernias]	Low
Trisomy 22	Mosaic Trisomy/ Non-Mosaic Trisomy 22/ Velocardiofacial syndrome	Low

Dubbed as one of the most common syndromes associated with CDH as well as congenital dysplasia of lungs and craniofacial anomalies in infants, Fryns syndrome is reported in up to 10% of cases with a diagnosed CDH.^13^ Among various overgrowth syndromes, Pallister-Killian syndrome and Simpson-Golabi-Behmel syndrome has shown consistent sonographic imaging that aids in the prenatal diagnosis of congenital diaphragmatic hernia.^18^

In the present case, the presence of fused earlobes and short stature (although individually, neither of them has significant value in direct genetic studies) warranted a syndromic approach to the autopsy, no significant morphological changes pertaining to congenital anomalies were noted other than the diaphragmatic hernia itself.

**Figure 2:**
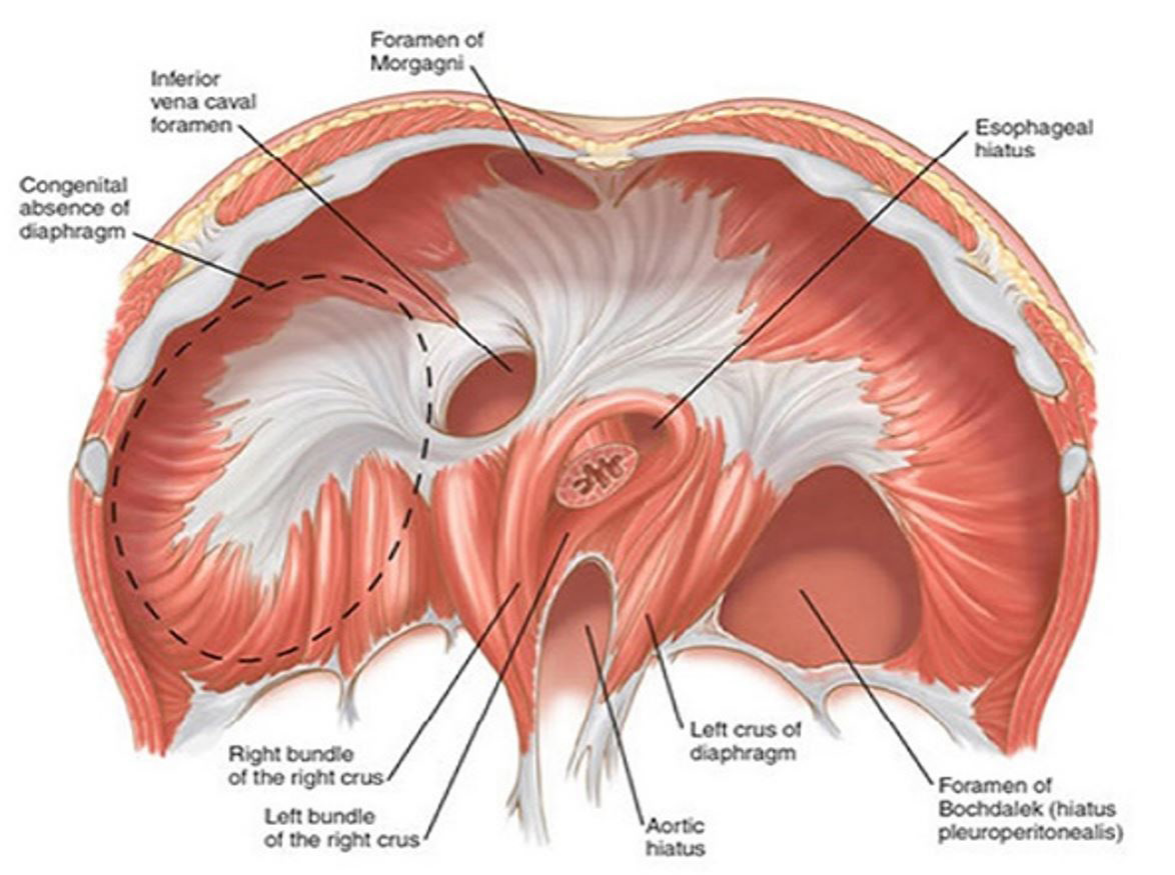
Foramina and congenital defects of diaphragm. Source: Haroun.^21^

Morphologically prevalent among the CDHs are “Bochdalek hernias”, where the posterolateral muscles of the diaphragm fail to fuse. If the same occurs in the anterior mediastinum towards the right side, it is known as Foramen of Morgagni hernia (Figure 2). Bochdalek hernias present almost exclusively in the pediatric age group, ranging from newborns to preschoolers, with only 5% occurring in adults.^5^ Despite their popularity, Bochdalek hernias have a low absolute incidence, with reports ranging from 1 out of 2,200 to 1 out of 12,500 live births; however, they are twice as common as Morgagni hernias.^22^^,^ 23

Males are more affected than females, with herniation occurring through the left hemidiaphragm in 70%–90% of the cases.^24^

Interestingly, a small foramen of Bochdalek may go unnoticed with spontaneous resolution, proving to be virtually harmless, whereas the wider ones could be fatal, as seen in the current case. It is only logical to assume that a small foramen can increase in size later in adult life, consequent to blunt trauma or exertion, which may explain the delayed presentation of a congenital diaphragmatic hernia. We believe was present in this case. Bochdalek hernias may remain dormant throughout childhood, only to be depicted incidentally on routine computed tomography scans.^23^

Studies and relevant literature reveal that the majority of adults with dormant CDHs who become symptomatic were subjected to some form of physical trauma.^2^^-^ 4 ^,^ 11 ^,^ 12 This trauma could be direct or indirect. The incidence of diaphragmatic injury occurs in up to 7% of those who suffer blunt abdominal/thoracic trauma and 3% to 15% for those with penetrating injury.^25^ This could lead to herniation through an existing defect or the formation of a new defect through which the abdominal contents can move into the chest cavity. Apart from blunt or penetrating trauma, other causes include physical exertion, sexual intercourse, and iatrogenic intervention, especially surgical intervention. It is believed that physical exertion causes a rise in intra-abdominal pressure, which is the underlying mechanism in the majority of hernias. This could range from engaging in physically demanding labor, or something casual like playing a game of tug-of-war.^26^ Although a recent review has shown a poor correlation of diaphragmatic hernia and increased intra-abdominal pressure seen in pregnancy, labor, and delivery,^27^ it was always believed they contribute to the non-traumatic incidence of CDH.^28^^,^ 29 Even something as trivial as sneezing, coughing or ingestion of a large meal have been reported to cause a diaphragmatic hernia.^29^

Early diagnosis has always remained an issue when it comes to catastrophes consequent to diaphragmatic herniation. However, recent years have seen an increment in the detection and treatment of many asymptomatic Bochdalek’s herniae, possibly due to a higher frequency of abdominal imaging.^5^ A study^29^ reported that among 13,138 abdominal CT scans performed in an institution over one year, 22 asymptomatic incidental Bochdalek hernias were found in adults, translating to roughly 0.17%. Usually, the stomach, large bowel or small bowel and omentum that usually herniates, but there are reports wherein spleen^30^ or a kidney^31^ gets dragged into the thoracic cavity. In the present case, we found omentum, along with the gastric fundus and a loop of the gangrenous small intestine in the thoracic cavity.

One of the key differences between the diagnoses of CDH in various age groups lies in its presenting symptoms. Neonatal CDH presents as pulmonary problems due to overcrowding of the thoracic cavity. On the contrary, in older children and adults, gastrointestinal pain or discomfort forms the chief complaint, reflecting the features of obstruction more often than respiratory complications.^32^ Nonetheless, Kato et al.^33^ reported the death of an adult due to collapse of the lung consequent to CDH, who presented with pulmonary symptoms. In the present case, the chief complaints were gastrointestinal, and we see a predominance of gastrointestinal symptoms over respiratory symptoms.

The adults presenting with a diaphragmatic hernia usually have a history of previous traumatic or physically strenuous events. In the present case, we found no evidence of trauma at the time of autopsy. Domestic abuse being common in the lower socioeconomic class, it is possible that an event of blunt trauma that occurred four months back could have caused the herniation. However, the husband denied the occurrence of any violent act upon questioning. Ingestion of a large meal was ruled out since the stomach contained fluid material only. There was no history of any recent pregnancies either.

However, physical exertion as a cause could not be ruled out. An initial episode of gastrointestinal symptoms 3 months back suggests a slow progression of the herniation. Since her occupation is that of a laborer, there are more chances that she was exposed to physical exertion, which is generally overlooked in the lower socio-economic class. It can be safely assumed that this may have been the root cause that precipitated the herniation.

Notwithstanding the etiology or presentation, prompt CT diagnosis could have saved the patient in the present case, assuming there was a rapid surgical intervention without postoperative complications. The fear of the COVID-19 pandemic led the patient to avoid hospital admission or routine investigations for her ailment, which was common during the initial wave of the SARS-CoV-2 infection. Hospitals recorded a major fall [up to 50%] in critical cases.^34^^,^ 35 The internet is also flooded with misinformation, which has caused an increase in cyberchondria-related anxiety and stress.^36^ Situation in India regarding fear and restrain from seeking medical help was similar.^37^^-^ 39 Such high levels of anxiety and unwarranted fear towards pandemics restrain people from availing healthcare facilities, leading them to death where critical care was a requisite for survival.

## CONCLUSION

Most cases of CDH remain asymptomatic and present as incidental findings in routine investigations. As a routine, laparotomy or thoracotomy, or a combination of both, form the basis of diaphragmatic hernia repair. Recent advances in minimally invasive surgery have made laparoscopic repair a more favorable option.^3^

Despite the lack of advanced genetic or chromosomal tests in our institute, we recommended the deceased’s family undergo further evaluation that may lead to the identification of genetic anomalies, which usually requires locus-specific DNA probes, FISH assay or alternate techniques. Citing financial constraints, they refused to perform the same. Although forensic pathology in developing countries is struggling to keep up pace with modern advances, it is imperative to implement protocols regarding collection of genetic data from all confirmed cases of congenital diaphragmatic hernia to gain clarity into the genetic causes as well as modes of inheritance of severe fetal anomalies such as CDH. Such a move would require legislative measures and funding from the government’s side. Alternatively, the government could commission private labs to analyze and report on the autopsy samples to expedite the otherwise slow process of awaiting the much-desired results.

In the present case, although CDH-associated anomalies didn’t seem to play a major role in death, it is safe to assume that if the excessive fear towards COVID-19 pandemic was eliminated, the female in question would have proceeded with the investigations and, assuming that a diagnosis was established, prompt surgical intervention could have prevented the fatal outcome. On a related note, not only is the government responsible for ensuring proper awareness of lethality pertaining to COVID-19, but also it is essential to curb disproportionate fear of a pandemic. This can be achieved by the dissemination of appropriate information and encouraging the citizens to seek help for any medical conditions that may affect them. An audit across the country for similar incidents focusing on deaths due to treatable medical conditions is also strongly recommended to understand the indirect effects of the pandemic.
